# Evaluation of first-principles Hubbard and Hund corrected DFT for defect formation energies in non-magnetic transition metal oxides†

**DOI:** 10.1039/d4ra07774a

**Published:** 2024-12-10

**Authors:** Daniel S. Lambert, David D. O'Regan

**Affiliations:** a School of Physics, SFI AMBER Centre and CRANN Institute, Trinity College Dublin, The University of Dublin Ireland Lamberda@tcd.ie

## Abstract

Recent advances have shown that first-principles DFT+*U* techniques, such as DFT+*U*+*J* with parameters from linear response, are capable of high bandgap accuracy in transition metal oxides at a fraction of the computational cost of hybrid functionals. Extending the use of these functionals to defect calculations could save computational resources, but there is limited knowledge on whether such techniques are capable of reliably modelling defect energies. Furthermore, the use of separate *U* and *J* values for the same atomic species in different chemical environments, within the same system, can introduce significant errors into formation energy calculations. In this work, for ZrO_2_, HfO_2_, and TiO_2,_ we compare calculated defect properties for PBE, DFT+*U*+*J*, and prior results from the literature. For ZrO_2_ and HfO_2,_ we identify three different practical methods that address the environment-dependent *U* and *J* value problem acceptably well, unlike the default naïve approach that yields unphysical defect formation energies. Our proposed techniques all yield formation energies, transition levels and defect concentration predictions that, while not identical to each other, are qualitatively in line with literature values. In TiO_2_, the formation energies are reasonably accurate, yet the localisation behaviour differs from that of the most reliable literature comparators, indicating a remaining difficulty for DFT+*U*+*J* with shallow defect levels.

## Abstract 2

A cost-effective and fast DFT+*U*+*J* technique qualitatively matches with expensive simulations for defect simulations.

## Graphical abstract

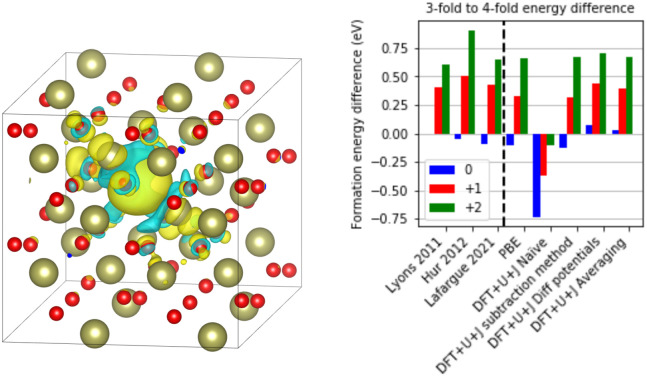

## 1. Introduction

The modelling of defects using density-functional theory (DFT) requires the use of large supercells, which take large amounts of computational time to simulate. This makes the use of low-cost approximate functionals such as PBE^1^ and LDA preferable to computationally expensive hybrid functionals such as HSE06 (ref. 2) and PBE0.^3,4^ However, the low-cost functionals mentioned often greatly underestimate the bandgap of the material on the basis of the Kohn–Sham eigenspectrum.^5^ This, in turn, reduces the reliability of defect energy properties such as formation energies, which depend on electronic transition levels.

In order to correct the bandgap of materials without resorting to the use of hybrid functionals, and possibly even to improve upon hybrid functionals, *in situ* corrective methods can be used, such as in a family of methodologies employing the Hubbard *U* parameter, known as DFT+*U*.^6–8^ In its simplest form, DFT+*U* adds an energy term that tends to penalize partial occupations of localized orbitals. The magnitude of this correction is set by a fixed “*U*” parameter, which can either be chosen on an empirical basis or calculated from first principles with a technique such as the finite-difference linear response method^9–12^ or minimum-tracking linear response method.^13,14^ The study of defects, particularly vacancies, in oxides using DFT+*U* has become increasingly commonplace over recent years, including some very sophisticated and successful studies.^15–17^

In addition to the Hubbard *U* parameter, Hund's *J* parameter is sometimes incorporated to improve the description of interactions between localized electrons of spin. The Hund's *J* parameter, within the corrective interpretation of DFT+*U*, has recently been associated with addressing static correlation error,^18^ which is an erroneous energy dependence on magnetization.^19^ Though counter-intuitive, this error can be substantial even in closed-shell systems where the magnetization itself vanishes. There are a variety of methods for incorporating the *J* parameter, including the widely-used Dudarev functional.^20^ In this work we primarily investigate a specific functional, referred to as “DFT+*U*+*J*” when originally proposed by Himmetoglu *et al.*^21^ (this is not the only functional sometimes labelled DFT+*U*+*J*). This functional takes the form of the Dudarev functional, plus an additional term proportional to the *J* parameter for interactions between opposite spins. Both the *U* and *J* may be calculated on the same footing through linear response techniques, albeit that in the case of *J* this is still rather novel and not commonplace. Particularly when using the convention for Hund's *J* as elaborated in Linscott *et al.*,^14^ DFT+*U*+*J* has been shown to provide very accurate bandgaps for MnO^14^ and TiO_2_.^22^ It should be noted, however, that the accuracy of standard DFT+*U*+*J* functional forms for total energies has recently been shown to be limited.^18^

In our previous work,^23^ upon which we build here, we showed that first-principles DFT+*U*+*J* provided a bandgap accuracy, over a test set of transition metal oxides (TMOs), that was similar (neglecting zero-point phonon and finite-temperature effects) to that of hybrid functionals such as HSE06. DFT+*U*+*J* was found to have a mean average error of 11%, the same as HSE06 and lower than the 15% error of PBE0. It was also demonstrated that DFT+*U*+*J* provides a lattice volume that is more accurate than the uncorrected PBE functional, with a mean average error of 2.33% compared to the 2.96% of PBE.^23^

For this work, we chose to study the widely-applied and well-characterized isoelectronic oxide series composed of ZrO_2_, HfO_2_ and TiO_2_, building on the bandgap results on each material from the previous work. Using DFT+*U*+*J* on these materials yielded bandgaps that were reasonably close to experimental values, with underestimation of the bandgap in TiO_2_, overestimation in HfO_2_, and an almost exact match with experiment in ZrO_2_. This set is a well-controlled one on which to judge the effectiveness of the DFT+*U*+*J* technique when applied to defect studies, because while changing little else it ranges from the very localised DFT+*U* projector orbital regime of 3d orbitals on Ti, to the rather diffuse one of 5d orbitals on Hf. At the same time, the closed-shell nature of these materials allows us to avoid certain ambiguities about how *U* and *J* are calculated (technically, the ‘simple’ and ‘scaled’ spin-polarized linear response schemes become equivalent) and, ideally for the present study, two of the oxides harbour oxygen atoms in different coordination environments. Thus any deficiencies in the processing of total-energy differences in first-principles DFT+*U*+*J* (with site-specific parameters) becomes very evident and even, as we will see, numerically catastrophic in these systems, while all other factors are relatively simple and well controlled.

For defect calculations, inaccurate bandgaps create problems for predictions of defect transition levels, because the defect levels may be predicted to be too shallow or even absent, for the same reasons that the bandgaps are underestimated.^24^ For materials with known bandgaps, defect studies often involve tuning the functional to the experimental bandgap by varying the *U* parameter, or varying the HSE screening parameter alpha,^25^ or using a direct scaling technique such as the scissor operator.^26^ These techniques are not first principles in nature, and so they cannot be applied to new materials or materials with uncertain bandgaps.

Defect formation energies can also be used to produce defect concentration predictions and Brouwer diagrams for intrinsic and extrinsic defects.^27,28^ These predictions are affected by material bandgaps as well as the magnitudes of formation energies, and will be more accurate when derived from simulations with both accurate bandgaps and accurate formation energy calculations. However, accurate defect calculations often require the use of large supercells that are computationally costly.^29^

The use of fast, first-principles bandgap correction methods such as DFT+*U*+*J* could address both of the latter issues, opening the path for much faster, accurate, high-throughput first principles screening of defect properties. However, the effect of DFT+*U*+*J* on defect calculations has not been studied extensively, much less how best to incorporate coordination-dependent *U* and *J* parameters in processes where the coordination changes.

Indeed, the use of DFT+*U*+*J*, and related DFT+*U* family functionals, for defect calculations provides some fresh challenges which are the key focus in this research. First-principles methods for calculating *U* and *J* can result in different parameters for inequivalent O atoms in different environments,^30^ since the parameters are as much a property of the chemical environment, as they are a property of the localized orbital shape. This poses no problems for bulk structure properties, but, as explained in Section 2.3, it raises significant issues for calculations of defect formation energies, due to the lack of error cancellation associated with absolute energies. This problem would also apply to other first principles techniques which yield separate *U* values for O in different environments, such as the DFT+*U*+V method that has been explored recently, *e.g.*, in Timrov *et al.*^31^

In this work, we propose three practical methods of compensating for the issue of differing *U* values. These all produce results that are qualitatively in line with previous research that did not encounter this issue. The comparability of DFT+*U* type total energies more broadly, with state-specific *in situ* calculated corrective parameters, is an open question with current evidence suggesting that it is also problematic, at least with currently commonplace functional forms. This is the case, for example, when spin-state specific parameters are calculated and the resulting total energies are compared.^32^ In that context it proved to be better to rely on a degree of cancellation of error, whereupon the total-energy differences proved to be very satisfactory.

In this work, the first-principles DFT+*U*+*J* method is used to perform defect calculations on three TMO materials, specifically rutile TiO_2_, monoclinic ZrO_2_ and monoclinic HfO_2_. In previous research, it was shown that these materials were reasonably well captured with DFT+*U*+*J*, with TiO_2_ underestimating, HfO_2_ overestimating, and ZrO_2_ almost exactly reproducing the experimental gap.^23^ The O vacancy defect formation energies are calculated in charge states 0, +1 and +2 for each material, for both the PBE functional and DFT+*U*+*J* based upon PBE, in order to compare their results for defect formation energies, defect transition levels, and electronic structures, and to investigate the feasibility of the DFT+*U*+*J* technique.

## 2. Methodology

### 2.1 DFT+*U*+*J* functional corrections

There are a significant number of variations in the use of Hubbard *U* corrections, which have found increasing success at capturing bandgaps from first principles.^33^ In this work, the label DFT+*U*+*J* is used to refer to a specific functional originating with Himmetoglu *et al.*^21^ The convention for defining Hund's *J* from Linscott *et al.*^14^ is used, and more specifically the self-consistent field variation of this previously used in our own ref. 23. Ordinarily, different linear response calculations are required for the Hubbard *U* and Hund *J* parameters. However for ultimately non-spin-polarized systems such as those investigated here (in their pristine bulk form), it is possible to reduce this to a single simultaneous linear-response calculation using the equivalent ‘gamma’ method,^22,23^ halving the required number of finite-differences calculations.

The DFT+*U*+*J* corrective functional used here is defined^21^ by1
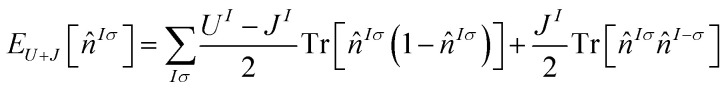
where *n̂*^*Iσ*^ represents the projected Kohn–Sham density matrix onto the orbitals that define the chosen subspace indexed *I*, for spin *σ*. The parameters *U* and *J* scale the magnitude of the correction. A further plausible ‘minority-spin term’ from^21^ is not included, following now-standard practice.

The values of *U* and *J* can be determined using linear response calculations. This can be achieved through by applying *α* and *β* perturbations, on a single representative target subspace *I* for a given chemical species only, to construct the free energy

2 *W* = *E*_DFT_ + *αN* + *βM*.

Here, *N* = *n*^↑^ + *n*^↓^, and *M* = *n*^↑^ − *n*^↓^, with *n*^↑^ and *n*^↓^ being the spin up and spin down occupancies, respectively (given by tracing the subspace density matrices).

The *U* value is calculated by determining the slope of the linear response of the occupation number *N* to the corresponding perturbation strength, in the self-consistent field formalism, before (*χ*_0_) and after (*χ*) screening takes place, per3
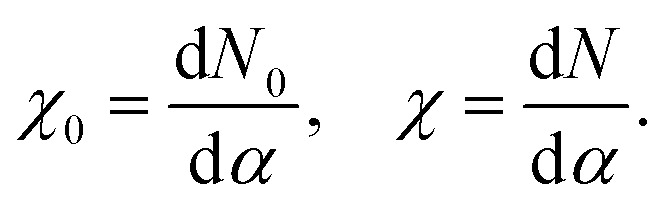


The *U* value is then calculated as

4 *U* = *χ*_0_^−1^ − *χ*^−1^.

The Hund *J* is then calculated in a similar manner by determining the bare and relaxed linear response of *M* to an applied *β*, per5
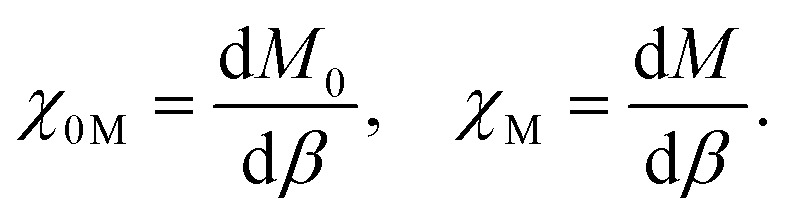
whereupon *J* is calculated as (noting the opposite minus signs to the *U* case)

6 *J* = −*χ*_0M_^−1^ + *χ*_M_^−1^.

The *U* and *J* values for TiO_2_, ZrO_2_, and HfO_2_ for each atom were calculated by the present authors in ref. 23, using supercells of 3 × 3 × 5, 3 × 3 × 3, and 3 × 3 × 3 multiples of the unit cell, respectively, in order to isolate the perturbed atom from its periodic images and to converge the screening environment. The linear response values found for TiO_2_, ZrO_2_ and HfO_2_ are summarised in Table 1. Since the 3-fold and 4-fold coordinated O atoms are inequivalent, the DFT+*U*+*J* linear response methodology yields different *U* and *J* values for the two types of O atoms in ZrO_2_ and HfO_2_.

**Table 1:** Table of *U* and *J* values (eV) for each of the three materials in this study from ref. 23, along with the averaged *U* and *J* value used in the “averaged O” method

Material	*U* (metal)	*J* (metal)	*U* (O_1_)	*J* (O_1_)	*U* (O_2_)	*J* (O_2_)	*U* (O average)	*J* (O average)
Rutile TiO_2_	3.24	0.38	11.24	1.70	n/a	n/a	n/a	n/a
Monoclinic ZrO_2_	1.74	0.34	14.28	2.34	15.45	2.56	14.86	2.45
Monoclinic HfO_2_	1.44	0.32	16.19	2.83	17.81	3.14	17.00	2.97

For reasons detailed in the next section, it can be useful for defect calculations to avoid having different *U* values for different O atoms, so an average *U* and *J* was also used in parts of this study, where emphasised. The large *U* values found for the PBE functional on oxygen 2p orbitals is noteworthy, albeit that those orbitals are more localized than the metal d orbitals here, when plotted.^23^ A detailed explanation of why large oxygen 2p Hubbard *U* parameters arise, in terms of chemical trends in the chemical hardness and electronegativity, is provided in Section III.B of (ref. 34).

A summary of the resulting bandgaps compared to experimental values was reported in our previous work ref. 23 and is shown in Table 2. The DFT+*U*+*J* bandgaps are reasonably close to the experimental ones, considering the low cost of the method once the parameters are in hand. We find a slight underestimate for TiO_2_, a larger overestimate for HfO_2_, and a falling within the experimental range for ZrO_2_. For this study, the bandgaps for HfO_2_ and ZrO_2_ were also recalculated using the “averaged” values for all O atoms.

**Table 2:** Table of bandgaps calculated with the DFT+*U*+*J* method using the calculated *U* and *J* values, compared to experiment, from ref. 23. The DFT+*U*+*J* bandgaps using “averaged O” methods for the O atoms for ZrO_2_ and ZrO_2_ are also included

Material	PBE bandgap	DFT+*U*+*J* bandgap	Averaged O bandgap	Experimental bandgap
TiO_2_	1.83	2.79	N/a	3.03
ZrO_2_	3.64	5.57	5.61	5.59
HfO_2_	4.20	6.39	6.48	5.78

It was also shown in our previous research that the use of DFT+*U*+*J* did not result in excessive geometric or electronic structure distortion, as long as oxygen 2p orbitals are treated on the same footing as metal d orbitals.^23^ This makes it more plausible that defect formation energies calculations would give reasonable results, as we now proceed to investigate.

### 2.2 Defect formation energy equation

The defect formation energy *E*^f^ can be calculated^35^ according to the equation:7
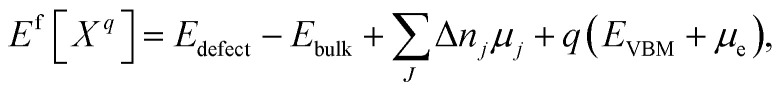
where *E*_defect_ and *E*_bulk_ are the DFT energies of the defective and bulk supercells, *E*_VBM_ is the valence band maximum (VBM) energy, taken from a calculation of the bandstructure of the pure cell, *q* is the charge of the defect, *µ*_e_ is the Fermi level relative to the VBM, Δ*n*_*j*_ refers to the number of atoms of type *j* that have been removed from the system (with positive Δ*n*_*j*_ referring to removed atoms, and negative referring to added atoms), and *µ*_*j*_ referring to the chemical potential of the atom that was removed or added.

The defect formation energy can be used for many predictions, such as of defect concentrations under real world conditions.^36^ It measures the thermodynamic (but not kinetic) obstacle to forming a defect in the material.

Defect calculations are usually performed in large supercells, rather than in the unit cell, in order to reduce the charge interaction between a defect interacting with a copy of itself in a neighbouring repeating cell.^29^ In order to better compare with previous results, for consistency we have neglected the charge correction term (which can be rather sensitive to the fine details of its parameters) and instead examined the convergence of energy with varied supercell size. This allows for an easier comparison with previous literature values that used a range of different charge correction techniques.

A comparison of chosen and larger supercells found differences in PBE 0/+2 transition levels (*i.e.*, the Fermi-level at which the defect formation energy plots for these charge states intersect) of 0.01 eV for TiO_2_ in a 2 × 2 × 3 supercell, against that in a 3 × 3 × 5 supercell. This result was (for the 3-fold O vacancy) 0.01 eV for ZrO_2_ (2 × 2 × 2 against 3 × 3 × 3), and 0.04 eV for HfO_2_ (2 × 2 × 2 against 3 × 3 × 3). This indicates that the resulting errors in transition levels due to lack of charge correction are likely to be small, compared to the large errors soon encountered.

### 2.3 The effect of differing *U* values

The defect formation energy is a measure of environmental barriers, and thus will depend on assumptions about the defect's surroundings, particularly through the choice of chemical potentials. In this study, the chemical potential of O, the *µ*_*j*_, is found by simulating the isolated triplet O_2_ molecule, in a large (10 × 10 × 10 Ang^3^) simulation cell. Different *U* and *J* values are applied to the O 2p orbitals of the reservoir O_2_ molecule, as we go on to describe. All results are given under the O-rich condition, that is assuming that the chemical potential of O in the TMO is equivalent to that in the reference single-molecule simulation.

Defect formation energy results rely on error cancellation between the DFT simulations of the pure cell, the defective cell, and the reference oxygen calculation. In DFT+*U* calculations, each atom that is assigned a Hubbard *U* value will introduce an associated amount of Hubbard energy into the calculation (as will Hund's *J* values in DFT+*U*+*J*). If an oxygen atom has a Hubbard energy associated with it in the defective cell and the pure cell, but not in the reference oxygen calculation, then it's possible that error cancellation will no longer occur to the same extent as a regular DFT calculation. In a typical DFT+*U* paper this is sometimes addressed by adding a Hubbard term with the same *U* value to the reference calculation, however in our case, there are two different Hubbard terms for the same element in the same calculation. This also applies to similar first principles techniques such as DFT+*U*+V, which also have differing *U* parameters for different O environments.^30^

If we neglect this issue and do not apply any Hubbard *U* to the reference potential, the resulting “naïve” defect formation energy for the charge neutral O vacancy defects can be calculated with the following equation derived from eqn (7):8
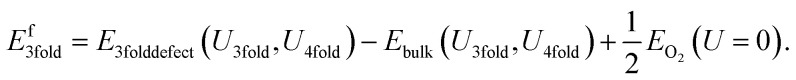
Here, *E*^f^_3fold_ is the formation energy of the 3-fold coordinated O vacancy, *E*_3folddefect_ is the DFT total energy of the defective supercell with a 3-fold O vacancy, *E*_bulk_ is the DFT energy of the pure cell, and *E*_O_2__ is the DFT energy of a reference oxygen simulation. In this equation and the next equations in this section, the numbers in brackets refer to the *U* parameter applied to the 3-fold and 4-fold O vacancies within the simulation, respectively, or in the case of the reference oxygen potential, the *U* parameter applied to every oxygen atom (for simplicity, only the *U* parameter is shown here, but for DFT+*U*+*J* the corresponding *J* parameters are also applied along with the *U*).

Similarly, the 4-fold charge neutral oxygen is given by:9
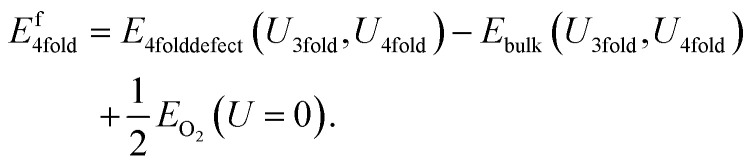
In our systems of HfO_2_ and ZrO_2_, the effects of a naïve treatment of this issue are most likely to be seen in inaccurate relative energies between 3-fold and 4-fold vacancies. The structures of HfO_2_ and ZrO_2_ have two different O atoms in two different environments, with one atom being 3-fold coordinated, another atom being 4-fold coordinated. As such, separate *U* and *J* values for each O type were calculated under the DFT+*U*+*J* method and applied, as shown in Table 1. However, as can be seen from eqn (1) and (2), the Hubbard correction energy is proportional to the chosen *U* and *J*. This means that the difference between a 3-fold and 4-fold O vacancy will be affected by the different corrections that apply to them. These differences can be quite large, in practice, and the Hubbard correction to the total energy using the ZrO_2_ 3-fold values is 10.0 eV per O, while that per O atom using the ZrO_2_ 4-fold values is 10.9 eV, a 0.9 eV per O difference.

In this research, we have devised and tested three plausible, practical methods to correct for the effect of the varying Hubbard energy contribution.

The first method, which will be called the “Differing potentials” method, involves using different O reference potentials for different vacancy calculations, with *U* and *J* applied to the reference potential simulation (the isolated molecule) matching the *U* and *J* values calculated from first principles for the O atom that is then removed from the solid. This means that the extra Hubbard energy of the O atom in the pristine supercell will be somewhat balanced out by the Hubbard energy of the O atom in the gas-phase reference calculation.

The equation for the differing potential method differs from the naïve method in only one respect: the reference oxide has a *U* correction applied, with the *U* value corresponding to the removed atom:

10 *E*^f^_3fold_ = *E*_3folddefect_(*U*_3fold_,*U*_4fold_) − *E*_bulk_(*U*_3fold_,*U*_4fold_) + 1/2*E*_O_2__(*U*_3fold_)

and for 4-fold defects:

11 *E*^f^_4fold_ = *E*_4folddefect_(*U*_3fold_,*U*_4fold_) − *E*_bulk_(*U*_3fold_,*U*_4fold_) + 1/2*E*_O_2__(*U*_4fold_).

The second method, referred to as the “subtraction” method, involves running the calculations as normal, using a molecular O_2_ reference calculation with no *U* or *J* applied but at the PBE level. The defect formation energy calculation is then modified simply by subtracting off the Hubbard energy correction in each simulation that has one. The Hubbard effect on the Kohn–Sham potential remains in place. This approach effectively treats the absolute energy of the correction as a spurious term to be subtracted out as a post-processing step, as is done with charge jellium energy in some charge correction techniques. The crystal structure and bandgap of the DFT+*U*+*J* method is preserved.

The equation for the subtraction method is:12
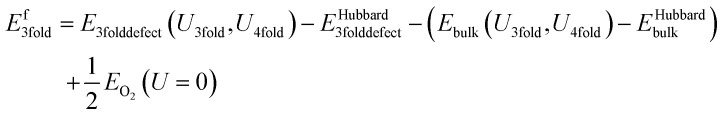


This is the same as for the naïve method, but two additional terms are introduced: *E*^Hubbard^_3folddefect_ is the total Hubbard energy in the defective cell simulation, and *E*^Hubbard^_bulk_ is the total Hubbard energy in the pure cell. Similarly, for a 4-fold defect:13
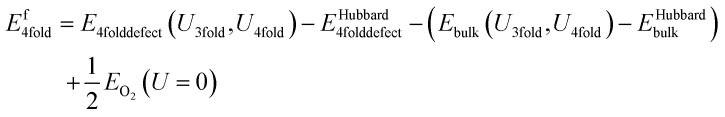


The third method is the “averaging” method, which is to average the O values for the 3-fold and 4-fold cases shown in Table 1 to obtain new values for *U* and *J*. These new values are applied equally to the 3-fold and 4-fold coordinated O atoms, as well as to the O_2_ reference gas-phase molecule simulation. The use of approximate *U* values may introduce extra errors in the simulations.

The averaging method has the following equation for a 3-fold defect:

14 *E*^f^_3fold_ = *E*_3folddefect_(*U*_avg_,*U*_avg_) − *E*_bulk_(*U*_avg_,*U*_avg_) + 1/2*E*_O_2__(*U*_avg_).

Unlike in the other methods, the *U* (and *J*) values used for 3-fold and 4-fold coordinated atoms are the same, averaged value.

15 *E*^f^_4fold_ = *E*_4folddefect_(*U*_avg_,*U*_avg_) − *E*_bulk_(*U*_avg_,*U*_avg_) + 1/2*E*_O_2__(*U*_avg_).

A key test of each technique uses the difference in formation energy between the equivalently charged 3-fold O vacancies and 4-fold O vacancies, which is thought to be already well described at the PBE level. In the case of PBE calculations with no *U* or *J* applied, the two defect calculations have the same energy expressions, with the only difference being that an O atom is moved. The difference in formation energies will be the resulting difference in simulation energies and will be independent of any errors in the atomic oxygen chemical potential.

This is not the case in DFT+*U*+*J*, where the removed O atoms in the 3-fold vacancy and 4-fold vacancy have different associated *U* and *J* corrections applied. Since the DFT+*U* Hubbard energy, for example, is directly proportional to the applied *U*–*J* value, any errors in the absolute Hubbard energy will not be cancelled out when comparing to two vacancies. Given a perfect DFT+*U*+*J* functional, population analysis, and method to compute the parameters, this issue may self-resolve, but it is not the case using the definitions in contemporary use. The three different methods we propose are expected to address this in somewhat different ways. The “differing potentials method” imposes an approximate error cancellation between a given O atom in the solid, and a coordination-dependent reference O energy. The “subtraction method” will remove all Hubbard terms, and thus eliminate the effect of absolute Hubbard energies, at the expense of self-consistency. The “averaging method” method balances out the extra Hubbard energy on each O atom irrespective of its environment.

### 2.4 Computational details

Calculations were performed with the Quantum ESPRESSO package version 6.6,^37^ using the PBE exchange correlation functional and the neutral-reference PSlibrary1.0.0 (ref. 38) ultrasoft pseudopotentials. The energy and force convergence thresholds for relaxations were 6 × 10^−5^ Ry and 10^−4^ Ry Bohr^−1^, respectively, a Fermi–Dirac smearing of 0.01 Ry was applied, and the Brillouin zone was sampled using a *Γ*-centered Monkhorst–Pack grid.^39^

As described in our work in ref. 23, the wavefunction and charge density cut-offs were chosen by convergence testing to less than 1 meV per atom variation. The wavefunction cut-off used was higher than typically used, out of caution for the robustness of results. The supercell sizes used for each material are summarised in Table 3. The *U* and *J* values were calculated in ref. 23 as described in Section 2.1 and summarised in Table 1.

**Table 3:** Simulation details for each of the three materials. Supercell sizes are specified as multiples of the unit cell

Material	Wavefunction energy cut off (Ry)	Charge density energy cut-off (Ry)	Supercell size	*k*-Points
Rutile TiO_2_	120	480	2 × 2 × 3	2 × 2 × 3
Monoclinic ZrO_2_	120	480	2 × 2 × 2	2 × 2 × 2
Monoclinic HfO_2_	130	520	2 × 2 × 2	2 × 2 × 2

### 2.5 Defect concentration predictions

Under thermodynamic equilibrium, defect concentrations can be predicted using the law of mass action:^28^16
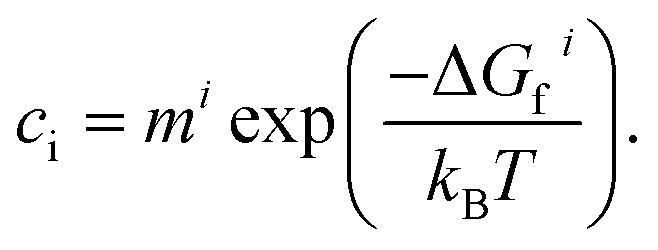
Here, *c*_i_ is the concentration per formula unit of defect *I*, *T* is the temperature, *k*_B_ is Boltzmann's constant, *m*^*i*^ is the multiplicity of the defect site, and Δ*G*_f_^*i*^ is the change in Gibbs free energy to form the defect, which we can approximate with the defect formation energy *E*_f_. This analysis only examines thermodynamic considerations and does not take into account kinetic barriers to defect formation.

In Section 2.2, the procedure for calculating defect formation energies was detailed. These formation energies are a function of Fermi energy within the material. At higher Fermi energies, there will be more negatively charged defects and free electrons, while at lower Fermi energies there will be more positively charged defects and holes, so there will be only one Fermi energy that yields charge neutrality, fulfilling the condition:17
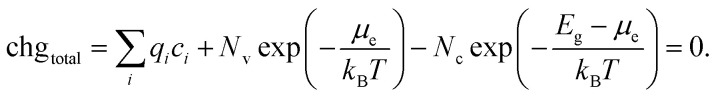
Here, *µ*_e_ is the Fermi energy relative to the VBM, *E*_g_ is the bandgap, *q*_*i*_ and *c*_*i*_ are the charge and concentration of each charged defect, *N*_c_ and *N*_v_ are the effective conduction band and valence band density of states (per formula unit), calculated using the parabolic approximation and the effective masses of electrons and holes under the low concentration condition:18
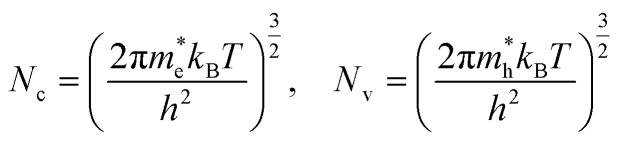
Here, 
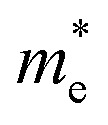
 and 
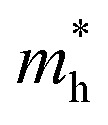
 are the effective masses of electrons and hole respectively, and *h* is Planck's constant. In this study we use the average of effective masses from PBE simulations as calculated previously in ref. 23.

The Fermi energy yielding charge neutrality can then be used to produce the final estimate for defect concentrations at that temperature. This can then be repeated for multiple temperatures to yield a graph of predicted concentration at each temperature. It should be noted that DFT is often unreliable when it comes to oxygen reference calculations,^28^ so the resulting defect concentrations will only be approximate.

## 3. Results

### 3.1 Monoclinic ZrO_2_ results

#### 3.1.1 Relaxed structure and electron localisation

**Fig. 1:**
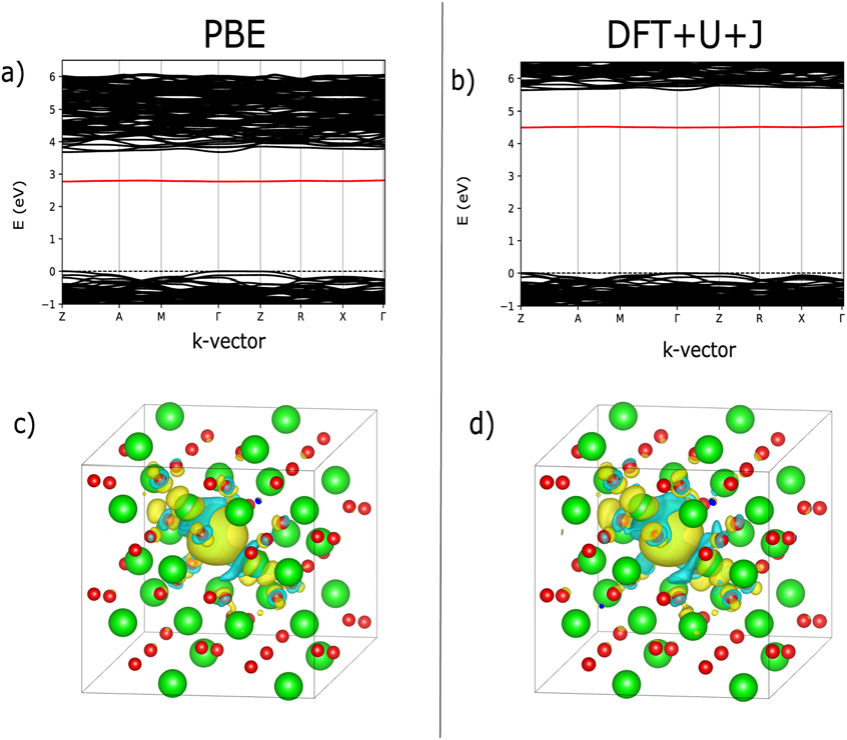
Comparison of defect bandstructure and electron localisation for the charge neutral 3-fold O vacancy in ZrO_2_. (a) Defect bandstructure for the PBE structure. (b) Defect bandstructure for the DFT+*U*+*J* structure. (c) Electron localization for the PBE structure. (d) Electron localization for the DFT+*U*+*J* structure. (c and d) Were calculated from the difference in charge density values between +0 and +2 calculations on the same +0 geometry.

Oxygen vacancy defects in ZrO_2_ were simulated in +0, +1 and +2 charge states, for both 3-fold and 4-fold O vacancies. To determine if the results were sensible, the localisation of vacancies was analysed. Fig. 1a and b show the band structure of the charge neutral 3-fold O vacancy defect, revealing a deep defect in the bandgap for both PBE and DFT+*U*+*J* simulations. Fig. 1c and d show the localisation of the excess electrons in ZrO_2_. To determine this, the charge density of the charge-neutral 3-fold O vacancy was calculated, and then the same calculation was repeated with the geometry fixed and two electrons removed from the simulation. Under the assumption that the excess electrons would be the first to be removed, subtracting one charge density from the other should indicate the localisation of the excess charge (at least in the anti-adiabatic limit). In both the PBE and DFT+*U*+*J* case, the excess electrons are localised around the vacancy site, as well as on the neighbouring O atoms. The use of DFT+*U*+*J* does not appear to have noticeably distorted the localisation.

#### 3.1.2 Formation energies and transition levels

For both the 3-fold vacancy and 4-fold vacancy case, the defect formation energy was calculated according to eqn (7) as a function of Fermi level for the +0, +1, and +2 charge conditions, for both PBE and DFT+*U*+*J*. However, as described in Section 2.3, the use of differing *U* and *J* values for differently coordinated O atoms results in problems with formation energy calculations.

**Fig. 2:**
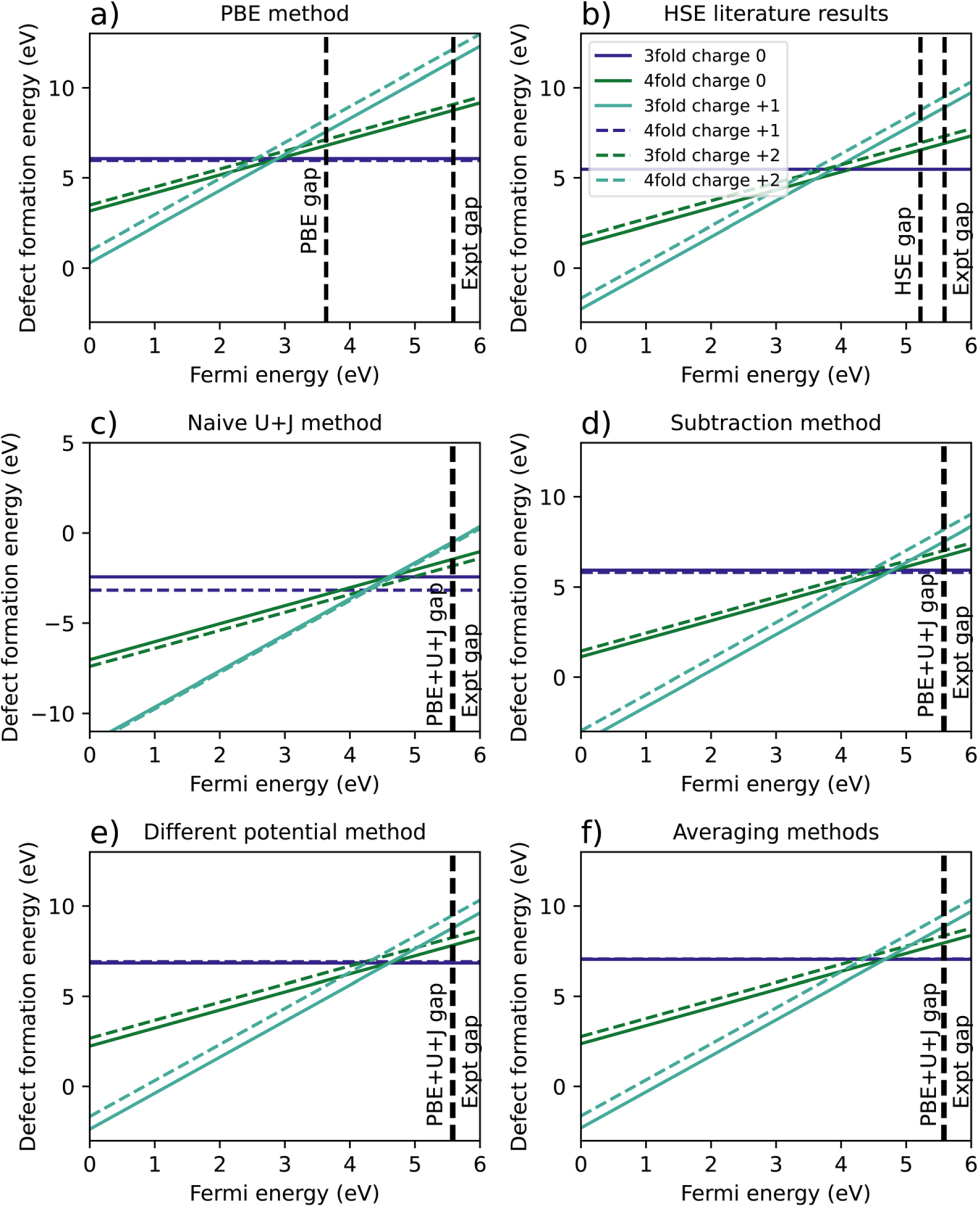
A comparison of calculated defect formation energies for ZrO_2_ under different methodologies. (a) Shows the regular PBE approximation, (b) is from Lyons *et al.*^40^ (c) refers to a naïve DFT+*U*+*J* calculation without corrections for differing *U*+*J* values on O atoms, and (d–f) refer to three different methods of correcting for these *U*+*J* values.

Fig. 2a shows the results of the PBE calculation, where the 3-fold vacancy is favoured for the +1 and +2 charges, but the 4-fold vacancy is very slightly favoured for the charge neutral case. Fig. 2b is a recreation of the results of Lyons *et al.*,^40^ in which the results were calculated using HSE with an hybrid-functional screening alpha parameter of 0.29. Just like in the PBE case, the 3-fold vacancy is favoured. Note that in the those results, the +1 charge is favoured for a very narrow range of Fermi levels. This qualitative difference may plausibly result from the mentioned authors having used a charge correction technique, *versus* our reliance on a large supercell.

Fig. 2c shows the original calculation result for DFT+*U*+*J*, with no corrections for differing *U* and *J* values. The naïve treatment predicts that the 4-fold O vacancy is preferred for all charge states and Fermi energies, including the +2 state, which disagrees with both PBE calculations and all previous literature findings^40–42^ to our knowledge. Fig. 2d, e and 2f show the results of the three different methods proposed to compensate for this effect, as described in Section 2.3.

The necessity of using corrections can be most clearly seen in the difference in energy between 3-fold and 4-fold O vacancies. In the PBE simulation and the HSE results from Lyons *et al.*,^40^ the 3-fold defect is clearly favoured for +1 and +2 charged defects, while the charge neutral defect formation energies are very close for 3-fold and 4-fold coordinated O defects. This also matches previous studies from Hur *et al.*^43^ and Lafargue *et al.*^42^ with the maximum charge neutral difference being 0.10 eV for the PBE calculation in this study.

The naïve method, without any corrections, yields completely different results, with the charge neutral 4-fold defect highly favoured over the charge neutral 3-fold defect by 0.74 eV, the 4-fold charge +1 defect favoured by 0.37 eV, and the charge +2 4-fold defect favoured slightly by 0.1 eV. For the reasons outlined in Section 2.3, these results are likely to be completely erroneous. All three error correction methods restore the results to be qualitatively similar to the PBE and literature results. The only difference from the literature methods is that the differing potentials and averaging methods slightly prefer the 3-fold defect in the charge neutral case, by 0.07 eV and 0.02 eV respectively, a relatively small discrepancy.

Table 4 summarises the location of the 0/2 transition level within the band gap, with the +1 charge state being ignored. The naïve method gives the closest result to the HSE reference for all three reference points, namely the VBM, the calculated conduction band minimum (CBM), or the experimental CBM. Of the three correction techniques, the differing potential method gives the closest results to the HSE reference for all three reference points. Overall, irrespective of the method used to treat the coordination-dependence of the calculated corrective parameters, the DFT+*U*+*J* method gives results that are much better in line with those of hybrid functionals than PBE, at a fraction of the computational cost after the parameters are once calculated.

**Table 4:** A comparison of the lowest-energy transition level for ZrO_2_, *i.e.*, the Fermi level at which the 0 and +2 charge formation energies take the same value. These levels are shown referenced to the VBM and to the calculated and experimental gap

Method	Band gap (eV)	0/2 Transition levels		
		Above VBM	Below calculated CBM	Below experimental CBM
PBE (3-fold)	3.64	2.89	0.75	2.70
Naïve DFT+*U*+*J* (3-fold)	5.57	4.61	0.96	0.98
Subtraction method (3-fold)	5.57	4.79	0.78	0.80
Differing potential method (3-fold)	5.57	4.61	0.96	0.98
Averaging method (3-fold)	5.57	4.67	0.90	0.92
HSE^40^ (3-fold)	5.22	3.87	1.35	1.72
PBE (4-fold)	3.64	2.50	1.14	3.09
Naïve DFT+*U*+*J* (4-fold)	5.57	4.29	1.28	1.30
Subtraction method (4-fold)	5.57	4.39	1.18	1.20
Differing potential method (4-fold)	5.57	4.29	1.28	1.30
Averaging method (4-fold)	5.57	4.35	1.22	1.24
HSE^40^ (4-fold)	5.22	3.57	1.65	2.02

**Fig. 3:**
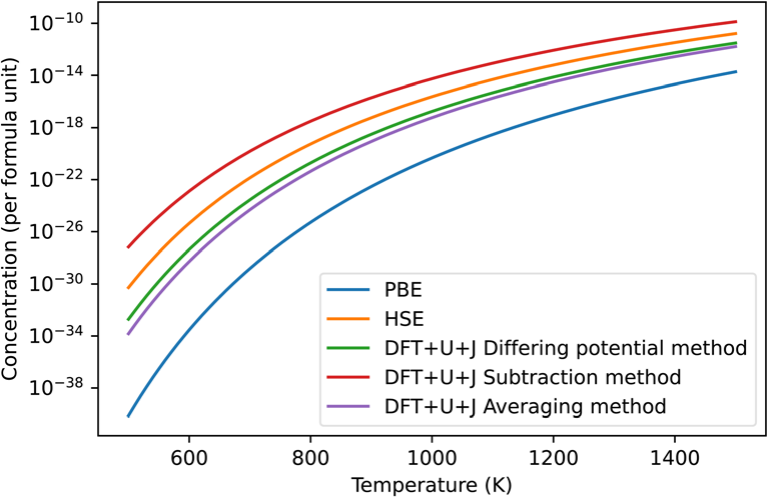
Predicted concentrations in ZrO_2_ of the dominant O defect for different temperatures, under all different techniques, under O rich conditions. The HSE results were derived from Lyons *et al.*^40^ The “naïve” method was omitted as it produced unphysical predictions. In all cases shown in the figure this dominant defect was the +2 charged 3-fold coordinated O vacancy.

The predicted concentration of O vacancies, under different temperature conditions, can be calculated from the defect formation energies using the method outlined in Section 2.5. It should be noted that these predictions are highly dependent on the reference chemical potential of oxygen. For simplicity sakes, and to match with Lyons *et al.*^40^ we have used a simulation of an isolated O_2_ molecule as reference. The results are shown in Fig. 3. The naïve method is not pictured as it gives unphysical results, predicting an oxygen defect concentration of over 100% at all temperatures. If taken seriously, this would erroneously imply that ZrO_2_ spontaneously breaks apart at room temperature. This provides more evidence that the naïve method is unreliable.

Of the remaining methods, the subtraction method gives the highest prediction of defect concentration. At 600 K, it predicts 3 orders of magnitude higher defects than the HSE method does, although this gap narrows at higher temperatures. The differing potentials and averaging methods give results that are almost identical in magnitude to each other. The results of these techniques are roughly 2 orders of magnitude lower than that of HSE at 600 K, making them 5 orders of magnitude lower than the subtraction technique. This difference reflects that the total Hubbard energy of an O atom in bulk ZrO_2_ is higher than that of an O atom in an O_2_ molecule. In contrast, the differing potential and averaging methods both give predictions that are within two orders of magnitude of the HSE predictions. Finally, PBE gives defect concentration that are much smaller than all the other methods tested, which is a reflection of its erroneously small bandgap for this material together the relatively shallow slope of the +2 defect formation energy with respect to Fermi level.

### 3.2 Monoclinic HfO_2_ results

#### 3.2.1 Relaxed structure and electron localisation

**Fig. 4:**
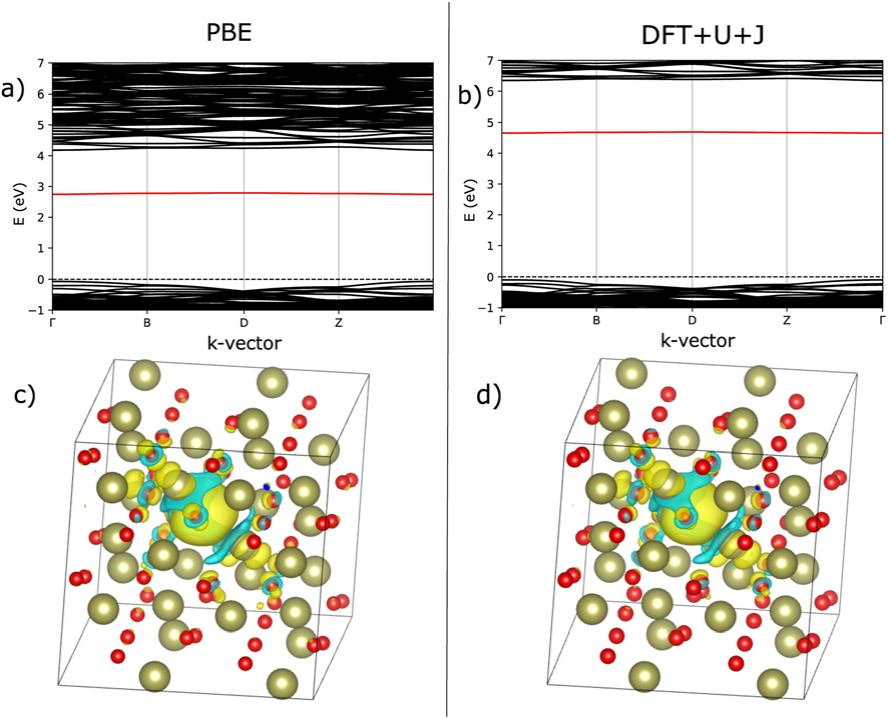
Comparison of defect bandstructure and electron localisation for the charge neutral 3-fold O vacancy in HfO_2_. (a) Defect bandstructure for the PBE structure. (b) Defect bandstructure for the DFT+*U*+*J* structure. (c) Electron localisation for the PBE structure. (d) Electron localisation for the DFT+*U*+*J* structure. Figures (c and d) were calculated from the difference in charge values between +0 and +2 calculations on the same geometry.

As with ZrO_2_, the 3-fold and 4-fold vacancies were simulated in each of three charge states, and the localisation behaviour was examined. Fig. 4a and b shows the bandstructure for both PBE and DFT+*U*+*J*, revealing the deep defect in the bandgap for both techniques. The use of DFT+*U*+*J* does not appear to significantly alter the nature of the defect, but it does succeed in opening the bandgap up to a reasonable value. Fig. 4c and d shows the localisation of the 3-fold O vacancies, obtained by calculating how much the charge density changes for the charge neutral O vacancy when two electrons are removed from the system, while fixing the ions. As with ZrO_2_, both techniques localise the extra charges around the O vacancy.

#### 3.2.2 Formation energies and transition levels

Similarly to the case of ZrO_2_, the HfO_2_ has two different O vacancies with different calculated *U* and *J* values, and hence a naïve defect formation calculation will result in erroneous results.

**Fig. 5:**
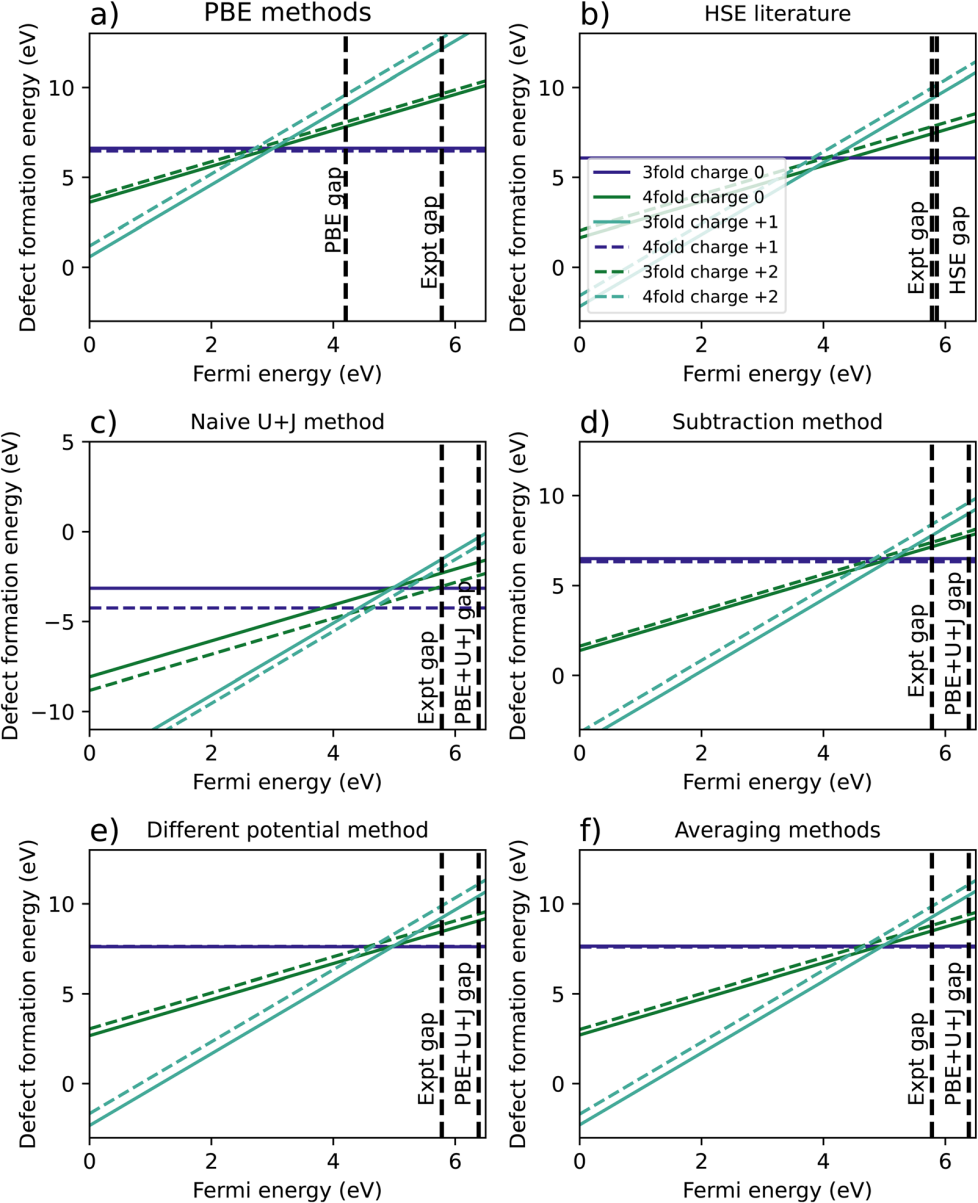
Calculated defect formation energies for HfO_2_ under 6 different methodologies. (a) Shows the regular PBE method, (b) is a from Lyons *et al.*^40^ (c) refers to a naïve DFT+*U*+*J* calculation without corrections for differing *U*+*J* values on O atoms, and (d–f) refer to three different methods of correcting for these *U*+*J* values.

Fig. 5 shows the resulting formation energy for a variety of methods, including a recreation of the results of Lyons *et al.*,^40^ using HSE. When it comes to the relative 3-fold and 4-fold energies, the resulting are qualitatively the same as was found for ZrO_2_, with the 3 fold vacancy being favoured in the +2 and +1 charge states for every methodology except the naïve *U*+*J* method, and the 3-fold and 4-fold vacancies having almost equal formation energies for every case except the naïve *U*+*J* methodology.

The difference between 3-fold and 4-fold coordinated vacancies shows a similar pattern to what was found with ZrO_2_. For PBE, the HSE results from Lyons *et al.*,^40^ and all three correction techniques, the 3-fold defect is favoured for +2 and +1 charged O vacancies, while the difference is small for charge neutral vacancies. The naïve method gives vastly different and likely erroneous results, clearly favouring the 4-fold defect under all charge states.

The main qualitative difference between non-naïve techniques is in which coordinated defect is favoured in the +2 state. The 4-fold defect is slightly favoured in the PBE, subtraction method and averaging cases, by 0.16 eV, 0.17 eV, and 0.05 eV, respectively. In Lyons *et al.*,^40^ both results were given as identical (to one decimal place), whereas in the differing potentials method, the 3-fold defect is very slightly favoured, by only 0.02 eV.

Table 5 summarises the location of the 0/2 transition level within the band gap. Unlike with ZrO_2_, in HfO_2_ the band gap is overestimated by the DFT+*U*+*J* technique. Again, the naïve DFT+*U*+*J* technique gives the closest answers to the HSE literature value, however as noted before it gives incorrect predictions in other respects. Of the corrected methods, the averaging method gives the closest result to the HSE values for all three reference points, with the calculated CBM reference point being the closest. As with ZrO_2_, the DFT+*U*+*J* correction transition levels are closer to the HSE results than the PBE is, for all three reference points.

**Table 5:** A comparison of the transition Fermi levels for HfO_2_ where the 0 and +2 charge formation energies are the same, referenced to the VBM and the calculated and experimental gap

	Band gap (eV)	Transition levels		
		Above VBM	Below calculated CBM	Below experimental CBM
PBE (3-fold)	4.20	3.02	1.18	2.76
Naïve DFT+*U*+*J* (3-fold)	6.39	4.97	1.42	0.81
Subtraction method (3-fold)	6.39	5.14	1.25	0.64
Differing potential method (3-fold)	6.39	4.97	1.42	0.81
Averaging method (3-fold)	6.39	4.97	1.42	0.81
HSE^40^ (3-fold)	5.80	4.13	1.67	1.65
PBE (4-fold)	4.20	2.64	1.56	3.14
Naïve DFT+*U*+*J* (4-fold)	6.39	4.65	1.74	1.13
Subtraction method (4-fold)	6.39	4.75	1.64	1.03
Differing potential method (4-fold)	6.39	4.65	1.74	1.13
Averaging method (4-fold)	6.39	4.65	1.74	1.13
HSE^40^ (4-fold)	5.80	3.83	1.97	1.95

**Fig. 6:**
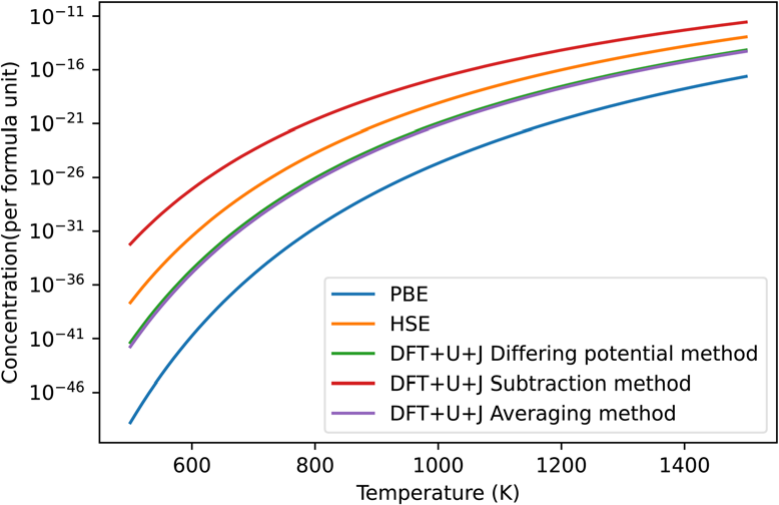
The predicted concentrations under different temperatures of O vacancy defects in HfO_2_ under O rich conditions. The HSE results were derived from Lyons *et al.*^40^ The “naïve” method has been omitted for giving unphysical results. The dominant defect for all methods was the +2 charged 3-fold coordinated O vacancy.

The predicted defect concentrations under each technique are shown in Fig. 6. As was the case with ZrO_2_, the naïve method gives unphysically large results that predict spontaneous dissociation and was thus excluded.

Of the methods shown in the figure, the subtraction method again overestimates the defect concentration by a large amount compared to other techniques, while PBE underestimates the value. The differing potential and averaging method give results that are almost identical for this material, and are the closest to the HSE results of Lyons *et al.*^40^

### 3.3 Rutile TiO_2_ results

#### 3.3.1 Relaxed structure and electron localisation

We examined the formation of defects in rutile. The removal of oxygen leaves two spare electrons, with the location of these electrons being well studied in previous literature,^44^ with localisation on Ti atoms neighbouring the vacancy generally considered to be the correct result, based on EPR studies by Yang *et al.*^45^ This localisation is usually found in hybrid studies, and DFT+*U* studies with high *U* values, but not in PBE or low-*U* studies.^44^

**Fig. 7:**
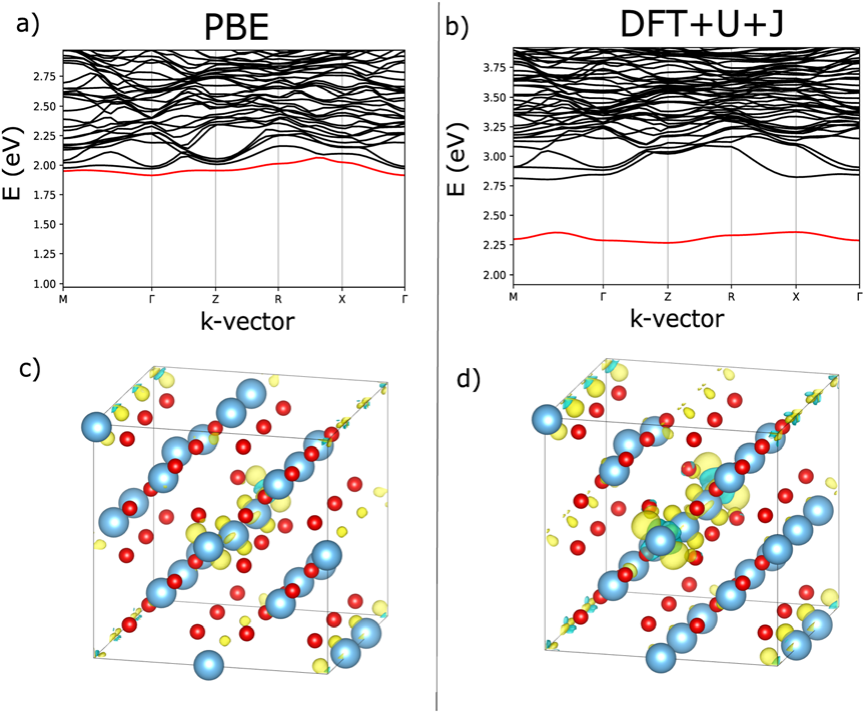
Visualisation of the electronic and localisation properties of the charge neutral O vacancy for rutile TiO2, using PBE or DFT+*U*+*J*. (a and b) Shows, for PBE and DFT+*U*+*J* respectively, the bandstructure of the defective cell near the CBM with the lowest defect band in the vicinity of the CBM highlighted in red. Figures (c and d) show, for PBE and DFT+*U*+*J* respectively, the difference in charge density for a relaxed defective cell between the charge neutral and charge +2 cases.

Fig. 7 shows an analysis of electron localisation for both PBE and DFT+*U*+*J*. Different initial magnetic configurations were attempted together with ionic relaxation in a fixed-volume cell, with some leading to higher-energy local minima, however for both functionals the high-spin, triplet-like magnetic configuration proved to have the lowest energy for the charge-neutral system. The supercell magnetic moment was 2.00 *µ*_B_ in both cases, while DFT+*U*+*J* provided a higher integrated absolute magnetization (of 2.85 *µ*_B_) than PBE did (2.55 *µ*_B_). Fig. 7a and b show the bandstructure of the defective supercell near the CBM, for PBE and PBE+*U*+*J*. The defect-related donor levels are majority-spin in character, and are somewhat dispersive in this supercell (*i.e.*, at this defect concentration) unlike in the case of the latter two materials. In the PBE case there is one only marginally distinct band, corresponding to double ionization of the defect. In the DFT+*U*+*J* case there is again only one well-separated donor level, corresponding to double ionization. This is less dispersive, and approximately 0.5 eV below the CBM. There is some evidence in the bandstructure of the entangled, again majority-spin single-ionization level at the conduction band edge.

Fig. 7c and d show, for PBE and DFT+*U*+*J*, with the defective cell structure frozen in its converged charge-neutral configuration, the charge density difference between the charge neutral case (with the excess electrons) and the case with two electrons removed (on the assumption that the localised electrons will be removed first). In a fully localised defect, this difference would be almost entirely localised as well.

In the PBE case of Fig. 7c, the electrons appear to have been mostly delocalised and spread much more uniformly among the Ti atoms throughout the bulk of the material, with a small preference for the Ti atoms near the vacancy site. In the case of the DFT+*U*+*J* calculation shown in Fig. 7d, the level of localisation appears to be somewhat increased yet with the same general profile, and some portion of the extra charge still spreads onto other sections of the cell. This is most similar to the “polaron” state described in ref. 46, but in this case and this supercell there also seems to be localisation on the Ti atoms further away as well. The localisation behaviour of shallow defects is only partially visualised by this methodology, which combines together the density for two different defect levels of rather different character.

#### 3.3.2 Formation energies and transition levels

**Fig. 8:**
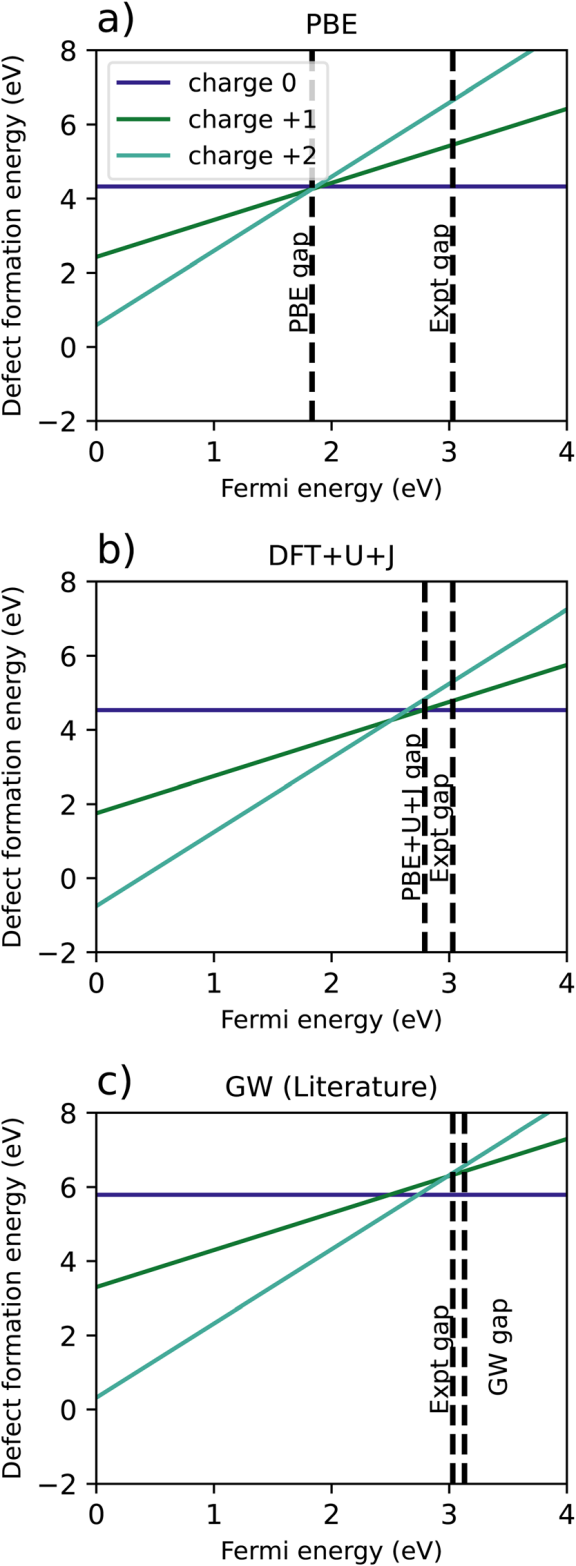
Comparison of defect formation energies for TiO_2_ calculated using (a) PBE functional, (b) DFT+*U*+*J* functional, (c) a recreation of G0W0 results from Malashevich *et al.*^47^

In the 2 × 2 × 3 supercell, for both PBE and DFT+*U*+*J*, defect formation energies were calculated for oxygen vacancies in the charge neutral, charge +1, and charge +2 states. Fig. 8 shows a comparison of each result, as well as a recreation of the GW simulation results from Malashevich *et al.*^47^ We note that it is questionable whether the formation energy results for the PBE case are physically meaningful, given the subsumed and shallow nature of the defect levels corresponding to single and double ionization, respectively. This is the case also for the single ionization level in PBE+*U*+*J*. In both cases there is qualitative consistency between the charge-neutral system bandstructures shown in Fig. 7 and the formation energy transition levels that may be read off from Fig. 8. We see in that for both PBE and DFT+*U* the 0/+1 transition level is at the CBM, reflecting the absence of distinct defect levels in the bandstructure. For the 1/+2 transition level is approximately at the CBM for PBE, but some 0.4 eV below the CBM in the case of DFT+*U*+*J*, again reflecting the distinct deeper (majority-spin) defect level the DFT+*U*+*J* bandstructure.

For PBE, and GW (which, unlike DFT+*U*+*J*, does not treat spin-symmetry broken solutions and resulting energy relaxation), there is no point in the where the +1 charge is favoured. It is further worth noting that the GW formation energy graphs are reconstructed from single quasiparticle energies, rather from total energies. In the PBE simulation, the +2 charge is the only charge favoured for the entire length of the band gap, with the 0, +1 and +2 charges all having almost exactly the same energy at the CBM. However, the introduction of DFT+*U*+*J* changes the relative formation energies of the charges, resulting in the +1 charge being favoured over a Fermi level interval near the conduction band edge.

Table 6 shows a comparison of the calculated transition levels, referenced to the VBM, the calculated CBM, and the experimental CBM. For all reference points, the DFT+*U*+*J* method yields the results closest to the GW method, while having a much smaller computational cost.

**Table 6:** A comparison of the 0/+2 transition Fermi levels for TiO_2_, that is where the 0 and +2 charge formation energies are the same, referenced to the VBM and the calculated and experimental gap

	Band gap (eV)	Transition levels		
		Above VBM	Below calculated CBM	Below experimental CBM
PBE	1.83	1.87	−0.04	1.16
DFT+*U*+*J*	2.79	2.64	0.15	0.39
GW	3.13	2.74	0.39	0.29

**Fig. 9:**
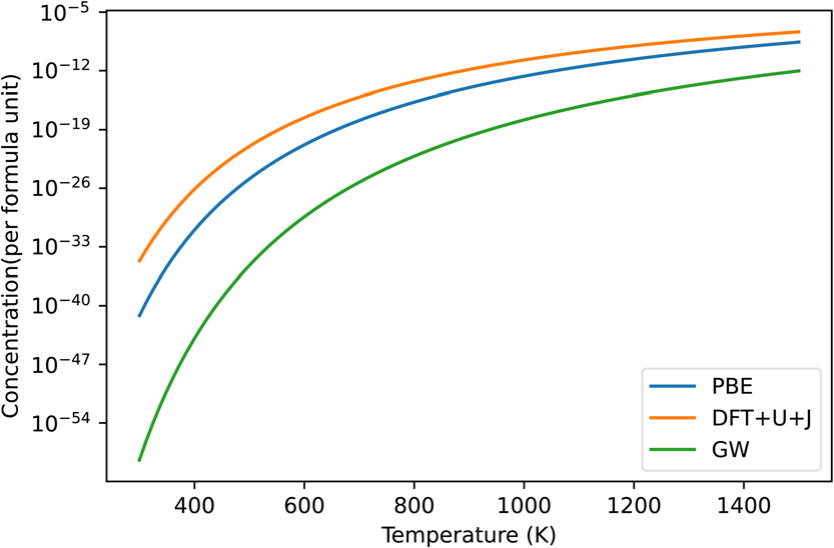
Predicted defect concentrations under different temperatures for TiO_2_ under O rich conditions. Only the dominant +2 charged O vacancy defect is shown. GW results are derived from the defect formation energies of Malashevich *et al.*^47^

Fig. 9 shows the predicted defect concentrations of O vacancies under DFT+*U*+*J*, compared to PBE and GW. In this case, the O potential is taken from a reference O dimer simulation with the same *U* and *J* applied as in the bulk simulation. This would correspond with either the “differing potentials” or “averaging methods” as used in HfO_2_ or ZrO_2_, which are equivalent since both O atoms have the same *U* and *J* value.

If we take the GW results as a benchmark, PBE overestimates the defect prevalence, with the DFT+*U*+*J* method overestimating it to a larger extent. This could be an indication that the inability to capture the shallow defect is resulting in an overestimation of defect formation energies. Alternatively, it could be an artifact of the differences in calculating O chemical potentials between this technique and that of Malashevich *et al.*^47^

## 4. Conclusion

In this study, the feasibility and effectiveness of using the DFT+*U*+*J* technique for calculating oxygen vacancy defect calculations were evaluated, using the test systems TiO_2_, ZrO_2_ and HfO_2_. For each material, the defect transition levels were also calculated and compared with literature values.

In ZrO_2_ and HfO_2_ the use of differing *U* values for the 3-fold and 4-fold vacancy introduced methodological problems for simulating such vacancies, due to the effect of differing Hubbard energies on defect formation calculations. This was seen most clearly in the gap between 3-fold and 4-fold energies, which is a valid total-energy difference quantity that should be accessible within DFT, where a naïve treatment leads to spurious results. This represents a special case of a general problem, at present, in the comparison of DFT+*U* type total energies when incorporating *in situ* calculated Hubbard parameters, such as in heat of formation, convex hull diagrams, or spin-flip energy calculations used to parametrise Heisenberg-type spin models.

Three different methods are proposed and tested for rectifying this situation, the “subtraction method”, “differing potential method” and “averaging method”. All three methods give 3-fold to 4-fold vacancy energy differences that broadly are in line with literature values, reassuringly, for ZrO_2_ and HfO_2_. Defect concentration predictions were compared for each method, finding that in both ZrO_2_ and HfO_2_, the naïve or uncorrected approach gives entirely unphysical results, while the differing potentials and averaging methods give results that match closely with reported HSE values. For these materials, the PBE approximation underestimates the defect concentrations, while the subtraction method overestimates them, as well as being an intrinsically inconsistent method.

Overall, we find that the use of at least some corrective method is crucial for certain results, such as predicting which defect is more dominant and predicting overall concentrations. For HfO_2_ and ZrO_2_, the subtraction method appears to fall short when it comes to defect concentration predictions, while the differing potentials and averaging methods perform similarly well on all numeric metrics. Overall we tentatively recommend the “differing potentials” method, which involves matching *U* and *J* parameters in the reference potential to those of the bulk material. This is because it may generalise better to systems with more greatly differing atom coordinations and/or Hubbard parameters, and almost certainly would do so when considering systems incorporating vacancies of more than one chemical species.

For TiO_2_ calculations with an oxygen vacancy, the defect transition levels based on the located spin-triplet neutral-system ground state matched reasonably closely with previous GW level work, both when compared to the valence and conduction bands. However, when first-principles DFT+*U*+*J* correction is applied, the +1 charge state is energetically favoured over a Fermi-level interval of 0.27 eV adjacent to the conduction band edge. A defect bandstructure analysis found that the DFT+*U*+*J* method separated the lower (spin-polarised) defect band from the conduction band minimum, whereas the PBE approximation doesn't achieve even this.

These results overall show that the first-principles DFT+*U*+*J* methodology gives defect formation energy results that are qualitatively similar to hybrid functionals at the task of placing defect transitions within a bandgap. While unlikely to be as accurate as hybrid results that are tuned to specific bandgaps, by construction, these results are completely from first principles and come at a fraction of the computational cost of hybrid functionals, making them potentially more suitable for large scale or high-throughput simulations. However, our results show that DFT+*U* type total energy difference calculations cannot be carried out naïvely, in the sense of neglecting the application of Hubbard correction (with at least some degree of consistency) across different systems contributing to the calculation. Ultimately, we anticipate that with the ongoing development of ever more comprehensive functional forms, population analysis methods, and parameter calculation methods for generalized Hubbard-corrected DFT, it may become unnecessary to take steps such as those described here to ensure viable results. Within the contemporary, ever more widespread practice of DFT+*U* for problems in computational materials chemistry, meanwhile, the proposed “differing potentials” method may prove helpful.

## Data availability

Our data is included in the ESI materials† of this submission. Specifically, the excel sheet “defects_supplementary” contains the results of the simulations for the three materials, as well as the reference calculations, and was used to prepare the tables used in the final projects. The jupyter notebook “Defect graphs and calculations” contains the code that generates defect formation energy diagrams from simulation outputs, as well as the band diagrams. The jupyter notebook “Brouwer calculations and graphs” takes the results from the defect formation energies and converts them into defect concentration graphs. An example Quantum Espresso pw.x input file for a defective ZrO2 calculation, “ExampleQeinputfile.in” is included to show how the DFT simulations were prepared.

## Conflicts of interest

There are no conflicts to declare.

## Supplementary Material

RA-014-D4RA07774A-s001
